# ‘SASA! is the medicine that treats violence’. Qualitative findings on how a community mobilisation intervention to prevent violence against women created change in Kampala, Uganda

**DOI:** 10.3402/gha.v7.25082

**Published:** 2014-09-12

**Authors:** Nambusi Kyegombe, Elizabeth Starmann, Karen M. Devries, Lori Michau, Janet Nakuti, Tina Musuya, Charlotte Watts, Lori Heise

**Affiliations:** 1 Department of Global Health and Development, London School of Hygiene and Tropical Medicine, London, UK; 2 Raising Voices, Kampala, Uganda; 3 Centre for Domestic Violence Prevention, Kampala, Uganda

**Keywords:** community mobilisation, primary prevention intervention, social norms theory, SASA!, violence against women, Uganda

## Abstract

**Background:**

Intimate partner violence (IPV) violates women's human rights and is a serious public health concern. Historically strategies to prevent IPV have focussed on individuals and their relationships without addressing the context under which IPV occurs. Primary prevention of IPV is a relatively new focus of international efforts and what SASA!, a phased community mobilisation intervention, seeks to achieve.

**Methods:**

Conducted in Kampala, Uganda, between 2007 and 2012, the SASA! Study is a cluster randomised controlled trial to assess the community-level impact of SASA! This nested qualitative study explores pathways of individual- and community-level change as a result of SASA! Forty in-depth interviews with community members (20 women, 20 men) were conducted at follow-up, audio recorded, transcribed verbatim and analysed using thematic analysis complemented by constant comparative methods.

**Results:**

SASA! influenced the dynamics of relationships and broader community norms. At the relationship level, SASA! is helping partners to explore the benefits of mutually supportive gender roles; improve communication on a variety of issues; increase levels of joint decision-making and highlight non-violent ways to deal with anger or disagreement. Not all relationships experienced the same breadth and depth of change. At the community level, SASA! has helped foster a climate of non-tolerance of violence by reducing the acceptability of violence against women and increasing individuals’ skills, willingness, and sense of responsibility to act to prevent it. It has also developed and strengthened community-based structures to catalyse and support on-going activism to prevent IPV.

**Discussion:**

This paper provides evidence of the ways in which community-based violence prevention interventions may reduce IPV in low-income settings. It offers important implications for community mobilisation approaches and for prevention of IPV against women. This research has demonstrated the potential of social norm change interventions at the community level to achieve meaningful impact within project timeframes.

Intimate partner violence (IPV) against women, defined as physical, sexual, or psychological harm by a current or former partner or spouse (1, 2), is a form of violence against women, a violation of women's human rights and a common experience worldwide (3, 4). Recent global estimates indicate that nearly one in three women will experience physical or sexual violence from an intimate partner during her lifetime (4). IPV is also a serious public health concern owing to its effect on women's physical, mental, and emotional health (5–8) and its association with increased HIV risk (9).

Traditionally, programmes to address IPV have focussed on establishing support services for victims and improving the response of formal institutions, including the police and health sector. More recently, emphasis has expanded to include efforts to prevent violence, and to address the factors that catalyse and sustain violence in relationships, families, and communities. One such innovative programme is SASA!, a community mobilisation intervention designed by Raising Voices and implemented in Kampala by the Centre for Domestic Violence Prevention (CEDOVIP), both of which are Uganda-based NGOs. SASA! uses positive, non-punitive programming which aims to change social norms and address the imbalances in power between women and men that perpetuate both violence against women and HIV (10). SASA! is currently being used by over 50 organisations in 15 countries in sub-Saharan Africa.

This paper seeks to convey the lived experience of SASA! and examine the pathways through which it appears to be influencing individuals, relationships, and community norms. It draws upon data collected as part of a 4-year evaluation of the SASA! programme conducted by the London School of Hygiene and Tropical Medicine (LSHTM) in collaboration with Raising Voices, CEDOVIP, and Makerere University.

## The SASA! approach

SASA's approach draws upon two theoretical frameworks: the Ecological Model and Proschaska and Velicer's Stages of Change theory (11). A key element of this theory is that individuals must pass through various phases before a new behaviour can be consolidated. SASA! embraces this notion and applies it to community-level change by creating a phased change process that takes communities through a structured programme of discovery, critical reflection, and skills building.

**Fig. 1:**
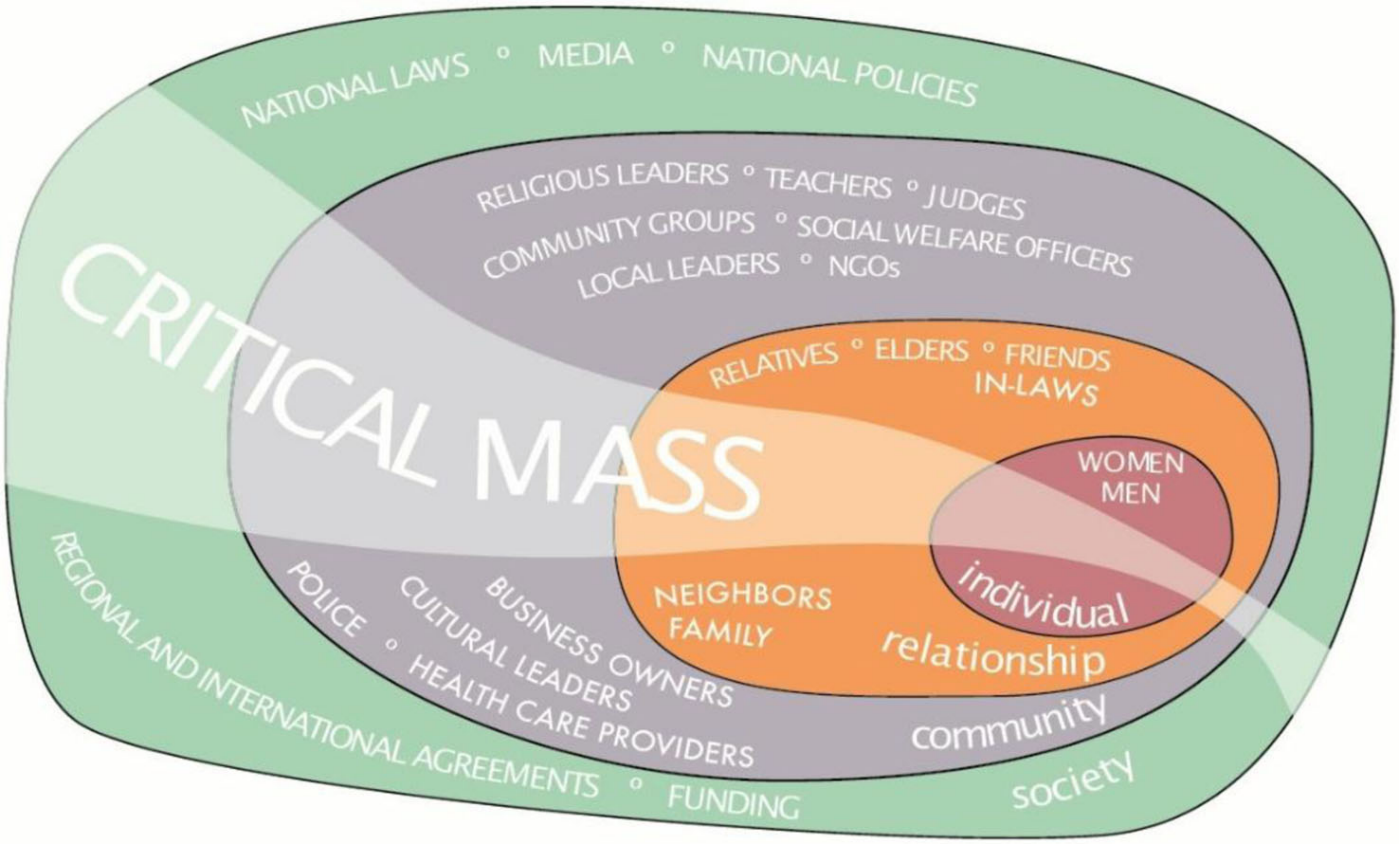
SASA! is part of the fabric of the community.

Depicted in Fig. 1, the Ecological Model (12) conceptualises IPV as the product of factors acting at the individual, relationship, community, and societal level (13). In so doing, it addresses individuals’ risk of experiencing or using violence as well as the norms, beliefs, and social and economic contexts that create the conditions under which IPV occurs (2, 13).

**Fig. 2:**
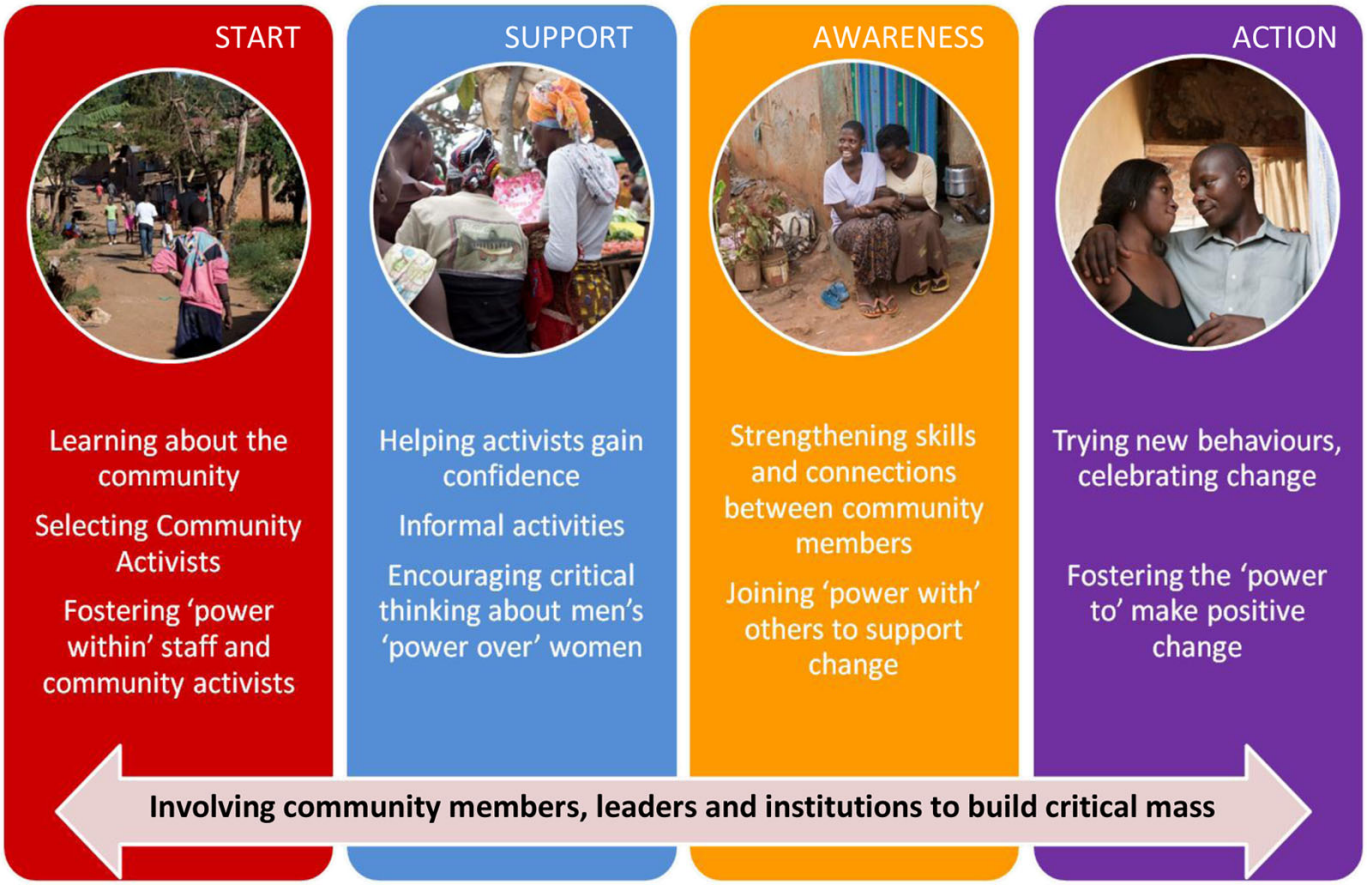
The SASA! approach: how it works.

Figure 2 illustrates the SASA! approach. In the **START** phase, CEDOVIP staff systematically learned about the community by mapping formal and informal resources and learning how communities were organised and structured. CEDOVIP also selected a number of community activists (ordinary women and men resident in the community). The emphasis of the **AWARENESS** phase was on helping activists to gain confidence to conduct informal activities within their communities. Community members were also encouraged to critically think about men's power over women and how this may manifest in their community. The **SUPPORT** phase was designed to strengthen the skills and connections between community members to encourage them to support those who were changing. This preceded the **ACTION** phase in which individuals were encouraged to try out new behaviours and celebrate change within their community. Throughout the phases, SASA! worked to build a critical mass by engaging a broad range of stakeholders.

SASA! employed multiple strategies. Through **local activism**, CEDOVIP staff supported community activists to conduct activities where people ordinarily congregated. The use of **media and advocacy** strategies by SASA! through small-scale media and street theatre also encouraged reflection and debate. Many SASA! activities were supported by a variety of contextually relevant **communication materials**. **Training activities** were offered to community activists to improve their confidence, skills, and ability to act as change agents within their community. The content of the various strategies evolved with each SASA! phase.

## The SASA! study

The SASA! study is a cluster randomised controlled trial to assess the impact of SASA!. Key aspects of SASA!, the study design (14), and the trial findings are available elsewhere (15). A future paper based on interviews with couples is also forthcoming. This paper reports on the findings of a qualitative study that was nested within the trial and involved 40 in-depth interviews with community members.

## Methods

### Study setting

The study was conducted in eight high-density impoverished communities in two administrative divisions in Kampala. Mobility in many parts of the study area was high. The majority of residents were self-employed in the informal sector with some study participants also generating income through letting accommodation or agricultural work outside the study area.

As elsewhere in Uganda, patriarchy – the concentration of both individual and institutional power in the hands of men – is a dominant aspect of the social-cultural context (16). Men are generally considered the head of the household and women are expected to be subservient to them. Kampala has a high prevalence of IPV and HIV. Of women aged 15–49, 52.3% are estimated to have experienced physical and/or sexual IPV (17), and 9.5% are estimated to be living with HIV (18).

### Sampling and data collection

Participants were sampled from individuals who responded to the follow-up survey of the SASA! trial. Criterion sampling – a form of purposive sampling – was used to select participants (19). To be eligible, community members had to report some exposure to SASA! and reduced violence in the last 12 months as compared to the period before. To maximise geographical spread, individuals were sampled from all SASA! intervention communities. Within these criteria, effort was made to maximise the heterogeneity of individuals’ characteristics (20) in order to capture the full range of participant experience.

In-depth interviews were conducted with 20 women and 20 men using a semi-structured tool. This included questions on: the genesis and evolution of their intimate relationship which was explored using a participatory tool; difficulties or challenges that they experienced in their relationship (including violence); their thoughts about SASA! and its impact on their intimate relationship; their views and understanding of violence against women in their community; and the impact of SASA! on their willingness and ability to act to prevent it. Mean interview duration was 90 minutes. Sex-matched interviews were conducted by a team of four research assistants who participated in intensive training for the qualitative study. This complemented their prior training as SASA! researchers. Participants were interviewed in a safe and private location of their choice, and all interviews were audio recorded. Of those who were invited to take part in research, two declined due to lack of time or interest. Six of the sampled individuals could also not be contacted, or an interview could not be scheduled during the period of data collection.

All participants provided written informed consent including permission for the interview to be audio recorded and for anonymised quotes to be used. Participants were provided with a referral list which included details of organisations that could be contacted for emotional, health, legal, or protection needs. Participants were also provided with the name and contact details of the SASA! community activist living closest to them. The study received ethical approval from Makerere University, the Uganda National Council of Science and Technology and LSHTM.

Participants ranged in age from 25 to 47 years old. The majority were Christian (Protestant or Pentecostal) and approximately half did not progress beyond primary education. Although not all participants were formally married to their partners, their relationship conformed to how marriage was understood in the context. In the results section, the terms ‘husband’ and ‘wife’ are used to denote participants’ intimate partners. All participants were parents and the majority had school-aged children.

### Analysis

The overall approach to analysis was thematic complemented by the use of constant comparative methods as described by Glaser and Strauss (21). Team meetings were held following each set of four interviews, and data and emerging themes were discussed. Any peculiarities in the data or novel lines of enquiry identified during these meetings were explored in subsequent interviews. Themes were also discussed with programme staff from Raising Voices and CEDOVIP in order to include their reflections in the on-going analysis process. The completed interviews were transcribed verbatim using a single-stage transcription process (22). The analysis of the interviews continued with an intense reading and annotation of the transcripts. Assisted by NVIVO 10 software (23), a constant comparative method of analysis was used to code and analyse the data through which a provisional coding frame was developed by the first author. Through on-going comparison of various properties in the data, this coding frame was finalised and included concepts that were determined a priori as well as those that emerged from the data. Through constant comparison, these concepts were further refined and compared to one another in order to develop a model to explain the pathways through which SASA! created change at the relationship and community levels as reflected in the structure of the findings below.

## Results

The description of the results presented below focusses on the relationship and community levels of the Ecological Model but, given that relationships are constituted of individuals, it also incorporates individual-level insights. We first describe the pathways through which those who experienced change in their relationship as a result of SASA! describe this change to have occurred before exploring how exposure to SASA! affected attitudes and responses to violence at the community level.

Whilst the findings focus on the pathways through which individuals experienced change in their relationships, not all relationships experienced the same breadth, depth, or degree of change. Some described, for example, that though they experienced a reduction in physical violence, other forms of violence, such as man's continued refusal to allow his wife to work outside of the home, persisted. Similarly, at the community level, not all participants felt compelled or able to act to support women that they knew or suspected to be experiencing violence.

### Effect on individual relationships

#### Encouraging mutual support

Most participants described how the challenges of daily life, including insecure and insufficient income and the high cost of living (particularly in the context of meeting the needs of children), exerted tensions on their relationship that led to quarrelling and disharmony. Conflict at times escalated into violence, particularly where women believed that their husbands were deliberately withholding money from them:

Poverty leads to violence because your partner will ask you for something and you may fail to provide it … the more she asks for things that you cannot provide the more she will improvise and get them from someone else, and when you discover that she got them from someone else, violence will ensue and in the end the relationship will end. (CM5, male community member)

This sentiment was expressed by many who also described how SASA! intervened to addresses these tensions by highlighting the benefits of working together for the good of their household. This provided a means for partners to renegotiate their gender roles and adjust their expectations of their spouse. This was achieved in two main ways. First, through SASA!, some men became more open to participating in domestic work to show support for their wives and to encourage ‘the development of their families’:

I had a big problem accepting certain things, for instance helping my wife at home with household work. I never believed in such things before … but they [SASA!] advised me to try and make sure we understand each other and things changed … they are good changes because you know, whenever you are two people … there should be agreement and you should be able to relate well, but if you don't relate well and everyone does their own things alone, it means there cannot be any development in the family. (CM6, male community member)

Although men's involvement was not always large, it was nonetheless appreciated by their wives as recognition of the many activities she conducted and an effort to relieve her of some of them, particularly when she was busy. This for many women was valued far more greatly than his actual contribution, suggesting that shifts in norms around the gendered division of housework were not of specific value or interest to women.

Men who were more actively and visibly involved in domestic work were considered relatively exceptional and were often viewed by the community as having ‘a good heart’ or having ‘been bewitched’ by their wives. In practice, these men were often younger or described how they had ‘grown up seeing their father doing housework’ and therefore did not view their involvement as extraordinary or demeaning. They contrasted with men who believed that ‘a man's place in the home’ was demonstrated by not participating in housework. As such, a small number of men rejected the suggestion that they should contribute to domestic work.

Amongst women, their husbands’ involvement in housework was regularly articulated as a marker of a good relationship or a period during which they were ‘relating well’. As such, men's involvement was interpreted as an act of benevolence which women encouraged by adjusting their behaviour in ways that conformed to gendered expectations. For some, often older women, or women who had no independent income, their husband's involvement in housework was not welcomed either because they ‘needed him to go out and look for money, not wash dishes’ or because they felt that supporting his involvement in domestic work was disrespectful or encroached on their role and domain of responsibility.

Second, through SASA!, many women reported being more willing to support their husbands by contributing to their households’ financial needs:

... As you know, women do not want to touch their money, they want to keep it for their personal use but they taught us in the [SASA!] activity that even when you have a husband it is good to support each other. They taught us that if your husband does not have money, then you should support him … when my husband does not have money I support him and it is not bad to, for example, buy bed sheets for your home. It does not make any sense to wait for your husband to buy bed sheets when you also have money … . In the past I would keep my money and I never wanted to touch it, but when we were taught, I agreed with what they taught us and now I am different. (CF3, female community member)

Whilst some women and men still expected the contribution to be repaid ‘when the man was able’, their contribution to household provision was noted by both men and women to reduce relationship tensions. For the majority of men, women's contribution was appreciated as a reduction in the pressure they felt to provide, and an acknowledgement by their wives of the challenges they faced in ‘looking for money’. For many women, it ensured that the family needs, particularly children's school fees, could be met in a timely manner, relieving them of the stress of having to secure money from their husband.

The result of these shifts in expectations was what many participants described as peace, and development within their households. Through this, participants reported more deliberate cooperation, especially to ensure that their children were at school and were well fed. The degree to which shifts in gender expectations towards more equitable relationship was evidenced was however contingent on the extent to which men were willing to reduce their control over their wives, particularly for women who were willing and able to work but were blocked by their husbands. Some husbands feared that this would precipitate their wife's infidelity, lead to her associating with ‘bad groups’, ‘getting a big head’ or no longer respecting him:

… well, it would not be bad if a woman helped in my responsibilities but you know it is not easy to trust a woman because you may let her work outside the family and then she gets a job. But as you know women admire a lot and so at work is where she might find someone to admire her and then change her mind into cheating. (CM6, male community member)

SASA! did however encourage this shift in some men through which they became more supportive of their wives working outside the home. For others, however, their willingness for their wives to work was predicated on their family's financial situation such that, had they been able to meet the household's needs, they would not have supported their wife's work outside the home. Women who worked outside the home were also still expected to be responsible for domestic chores and caring for children such that their absence from the home while working was at times a cause of relationship friction.

For a few women who did have an independent income, however, by challenging gender norms around women's right and ability to make financial decisions, SASA! played a role in improving their agency to spend their money as they wished:

… since I started participating in SASA! activities everything changed for the two of us. Since then we are more united and each one of us see the other as one person. No one complains about how much the other is spending their money… . I do not intervene in the way my wife spends her money. She spends it in the way she wishes to but I can no longer force her to use her money on anything against her wish. (CM3, male community member)

Not all women wanted to work, however, as they feared that if they had an income, then this would reduce their husband's willingness to provide for the family.

#### Improving communication, joint decision-making, and disagreement management

SASA! also played an important role in improving communication between partners around a variety of important issues. At the most fundamental level, SASA! encouraged them to discuss their likes and dislikes in order to ‘avoid doing things that would annoy the other’. Communication was often aided through the use of the SASA! materials that were distributed during activities and used by some participants as conversation starters, particularly for issues that were difficult to discuss.

For some, the role of SASA! in helping them to discuss financial issues was greatly valued, especially where it was done as a means of working together for the benefit of the family:

I don't hide anything from her … it was SASA! that taught us all that … They tell us that it is good for each one of you to inform the other about your income. You have to let them know how much you earn and together you decide how to use that money. You discuss what to buy, how much to save and you don't use another's money without their consent because you have power. You have to agree and if one of the parties does not want [to] then you don't force her. (CM3, male community member)

Allied to this was men's increasing willingness to vocalise the challenges they were facing in securing enough money for household needs. By communicating this to their wives, this helped her be ‘patient’ and relieved suspicions she may have had about his fidelity and expenditures. It also corresponded with women's increased willingness to contribute financially to household needs.

For some women, especially those who previously had little decision-making power, being able to discuss and make decisions together was a very important development. This included decisions around their children's schooling, where they lived, and the purchase of large items:

I got to know that everyone has a right to make a decision. [Before SASA!] I thought that decisions were made by the household head but now I know that everybody matters, including the children in the home. It's important to ask everybody how they feel about something before making a decision. (CF1, female community member)

Through being consulted and included in decision making, these women felt that their husband was showing respect and trust for them:

Truthfully speaking even in the area of respecting each other … I see that he respects me … I see that when I am talking to him … he gives me time and he listens to what I am saying … or he could even say that … . This is what I think … but what do you think about it, you see that he respects you. (CF19, female community member)

For some, this reflected an improvement in the esteem in which they were held and strengthened their sense of relationship security and desire to continue working together with their husband for the good of their relationship and their household.

For a few women, one of the most valued changes experienced as a result of improved communication was their husband's decreased insistence on sex:

… P: there are times when I would tell him that today I feel I do not want to have sex … he would then force me … and tell me ‘that is impossible, you came to my home, and this is the reason you came’ … but I went for SASA! and learnt about these things … he also got the opportunity … he watched a drama so he also got to know that a woman is not supposed to be forced to have sex … he has changed, he no longer forces me …

I: … How did that affect your relationship?

P: … when you tell a person that I do not want to have sex but he forces you, within you you feel disgusted with this person, and even hate him. But for a person whom you tell that today I don't want to have sex with you and he listens, you feel deep down within you that you have started to trust, love, and respect this person, because just by doing that, he has respected you. (CF19, female community member)

SASA! gave these women the confidence to start a discussion about sex which was not a subject they previously felt empowered to address, given their internalisation of dominant gender role norms and expectations. These discussions were most favourably received by men who also had direct and independent exposure to SASA! and thus had first-hand experience with an idea that was often new to them:

[from attending SASA! activities] I learned that some of the things I used to do were not right at all … for instance I thought that whenever I needed sex I had to have it without her denying me. I thought whenever I wanted sex, she would automatically want it. So whenever she would refuse, I would get so enraged and we would fight. (CM18, male community member)

Through acknowledging that there would be times of tension or anger between partners, SASA! also played an important role in equipping participants with practical, non-violent ways to deal with anger or disagreement. These strategies included encouraging people to apologise to their partner as ‘apologising breaks one down from anger so they become easier to deal with’. They were also useful for occasions when husband returned home drunk or late at night which was often when they were violent towards their wives. Women described how, prior to their involvement in SASA!, they would have confronted their husbands straight away. SASA! encouraged them to wait until their husbands were sober before discussing their concerns, as a strategy for reducing their risk of violence.

### Impact at the community level

#### Reducing the acceptability of violence against women

SASA! played an important role in raising awareness about different forms of violence and its consequences. For some women, it also helped to dispel their perception that violence was an act of love:

SASA changed me … I used to think that family secrets could not be revealed … [people also used to] say that if a husband does not beat his wife then she will not know that he loves her … all these beliefs were changed [through SASA!]. (CF19, female community member)

For the majority of female participants, SASA! also reduced the perception that violence against women was a private issue that women should endure, and helped many women to speak out about the violence they were experiencing:

Yes, most of the ideas [of SASA!] were new to us because most of us were in violent relationships … the men would do all sorts of things to us but we did not know that they were using violence against us … and we thought that a woman was not supposed to report anything that happens in the home … but then we learnt that we could report, or tell an outsider [about] things that happen to us in the home. (CF4, female community member)

Testimonies and discussion at community events were often powerful, and helped to challenge norms of silence around violence and further reduce women's perceptions that violence was a normal, if unpleasant, part of relationships:

Yes things have changed. You know when we go to the activities … you will hear people give testimonies of how their husbands have changed into good people. You will hear women say that my husband used to beat me but now he no longer does, or my husband used not to buy food for us but now he does. I think SASA! has brought a big change. (CF3, female community member)

Reduced acceptance of violence against women was also noted by several male participants, particularly those who had themselves used violence against their partner prior to their involvement with SASA! Overall when taken as a group, men reported reduced acceptance of all forms of violence against women including physical violence, demanding sex, deliberately not providing for their families, preventing their wives from working, and ‘barking at their wives instead of talking to them’. At the individual level however, not all men stopped using all forms of violence and control. Whilst many reported, for example, that they no longer slapped their wives, some still would not allow their wives to work outside the home.

#### Increasing support to women experiencing violence or men wishing to stop using violence

Prior to SASA!, the majority of participants described being reluctant to intervene for fear of being labelled a meddler given ‘people in Kampala keep to themselves’; fears for their personal safety; concern that intervention would lead to them being suspected of having an affair with one of the people involved; or that their support would not be welcomed. A few participants also described how they would have intervened only in the most severe instances of violence in order to prevent serious injury or death. Following their exposure to SASA! however, several participants reported increased willingness to act to prevent violence against women in their community and described how they had intervened at an earlier stage when a couple was ‘just quarrelling’ in order to separate them and give them a chance to ‘cool off’:

You have to respond as soon as you hear it because if you delay you might find that one has been hurt so much. Generally with violence there is no need to wait. It is rather better to intervene as soon as it happens. (CM3, male community member)

A few participants also described their increased willingness to talk to the couple and ‘counsel them about peace in their’ homes. For these participants, this was especially important because it gave them confidence that they had not only prevented immediate danger to the woman involved, but that she would be safer after they left.

Through engagement in SASA!, various community structures were also supported to provide better services to survivors of violence, and men who wanted to stop using violence. These included the Local Council structure (the most decentralised level of local government), other community leaders, health centres, and the police. For a few participants, exposure to SASA! was also important for improving the advice that they gave to women who were experiencing violence, especially where previously they had blamed them for the violence they were experiencing or told them ‘to do as their husband said’ in order to avoid the violence that they were experiencing.

Some participants did not feel able to intervene themselves however, but instead reported the violence. In response to persistent or serious violence, they often reported to the police in an effort to end the violence and have the man held responsible for his actions. In relation to what they considered ‘less serious violence’, women in particular reported to individuals in the community who they believed ‘were responsible for tackling violence’. They included SASA! community activists and members of the Local Council. In a similar way, many participants who intervened, referred women who they observed were experiencing violence to these structures (or to SASA! activities) in order that they may receive information or support. These resources were also used by women themselves as an important source of support:

I went to the women's representative in the community, she also works with SASA! I explained to her that I had persevered for two years ... I always felt ashamed to speak out, that he was beating me but I told her. By the time I told her it was too much for me. I had even lost the peace I had, the man was beating me regardless of day or night … now this lady summoned him and we sat, even my brother was there. They counselled him and the *nabakyala* [women's representative] told him about the SASA! activities and that you are supposed to talk to your fellow adult and not beat them … then my husband started changing. (CF19 female community member)

The presence of individuals in the community that had been trained by SASA! and could provide on-going and one-to-one support to partners was especially valued by women who had attempted to seek support from their families without success. The intervention of SASA! activists was often reinforced when their husbands agreed to attend SASA! activities, to read SASA! materials or to speak to other community members who were exposed to SASA! Indeed, a few actively engaged male participants, described how through SASA!, they were more willing and able to speak to other men about the violence they were using:

Normally after the activity as men we also meet the fellow man [who is using violence] and talk to him and stop him from doing what is wrong. It also gives him a chance to realise that what he has been doing has been hurting his wife and in most cases that is when most men change and convince themselves never to repeat certain things they have been doing. (CM3, male community member)

These men often used their own relationships as an example and highlighted the benefits that they and their partners were enjoying as a result of their no longer using violence. These included ‘peace and development in the home’, improved trust, and a sense of ‘being together with their friend’. Overall, participants believed that the reductions in violence that they had observed in their community were real especially as, given the housing density of their communities, they were able to observe directly the actions of their neighbours.

## Discussion

This paper provides evidence of the ways in which community-based violence prevention interventions may reduce IPV in low-income settings. The findings focus on the relationship and community levels of the Ecological Model with some insights on individual-level processes of change.

At the level of relationships, SASA! has encouraged many to explore the benefits of less rigid gender roles as a means of improving their relationship and household outcomes. The findings suggest that the loosening of gender-role strictures reflects an outgrowth of partners’ improved respect, desire to cooperate and support one another, rather than an ideological shift towards more expansive gender roles. In a similar way, by encouraging communication and joint decision-making, SASA! has also highlighted the benefits of mutual trust and respect between partners which many participants credit with having led to increased collaboration, relationship security and intimacy. This has also been important for reducing instances of violence which, together with learning about non-violent ways to deal with anger or disagreement, is valued as an important means through which relationships have become more peaceful.

At the community level, SASA! has played a fundamental role in increasing participants’ awareness of different forms of violence and its consequences. It has also reduced the acceptability of violence against women by fostering a climate of non-tolerance of violence. Amongst women, SASA! has challenged previous norms that women should not talk about the violence they are experiencing. Amongst men, SASA! has encouraged two important developments. First, it has generated and supported an important reference group constituted of ordinary men who no longer use violence against their wives. Second, this group has acted as an important source of support for men who wish to stop using violence by providing personal testimonies and encouragement.

Along with non-tolerance of violence, SASA! has also challenged the social norm that constrained individual's willingness to involve themselves in other people's business. This in turn has contributed to an emerging norm that individuals should act to prevent IPV in their communities. At a practical level, this has also required people to overcome concerns about the potential negative consequences of their intervention including the risk to their own personal safety. Nonetheless, SASA! has successfully encouraged some participants to act in response to the violence they observe. The role of SASA! in improving community-based structures and services has also cultivated greater support for sustained action and community mobilisation against IPV.

## Limitations

This research has a number of limitations. The findings reflect the experience of a particular group of people, namely those who were exposed to SASA! and reported that they had experienced change in relationship violence. By focussing on people who experienced change, we are unable to adequately reflect upon the limitations of the SASA! approach or factors that may constrain change at the individual, relationship, or community levels. The data on relationship-level change also only reflects the perspective of one member of the couple. This limits our ability to explore whether their partners would recognise the same changes in their relationship and ascribe these changes to SASA!. A forthcoming publication from the SASA! study does however provide data from both members of a couple and thus provides deeper insights into processes of relational change as a result of SASA!.

Contextual limitations also arose from the fact that during SASA! implementation, campaigning and political disturbances around the 2011 national elections resulted in a suspension of activities. This meant that the 4-year period over which SASA! was implemented equated to 2.8 years of SASA! programming, suggesting that SASA! may not have been optimally implemented.

The study also has a number of methodological limitations. Given the constraints imposed by the trial, the implementation of SASA! was somewhat truncated. To prevent contamination of control communities, community activists had to respect strict geographical boundaries when conducting activities. Similarly, religious institutions, which often cover wide population centres, were also not engaged. For a intervention that relies of diffusion of ideas, this presented an unnatural constraint which may have acted to reduce the community-level impact of SASA!.

A further methodological limitation is that all the interviews were conducted at follow-up and as such provide cross-sectional data. Given that the research sought to examine change over time, multiple interviews with the same individuals would have been valuable. This would also have provided insights into how participants experienced and negotiated the new ideas introduced by SASA!. It would also have meant less reliance on participants’ recall of their experiences which may have been coloured by desirability bias and the particular outcomes that they experienced in their relationship.

## Implications

The findings offer five important programmatic and contextual insights for community-based IPV prevention interventions and interventions that seek to change gender relations. First, this research demonstrates the potential for achieving meaningful behavioural and attitudinal change within project timeframe, indicating that social norm change does not necessarily need to be a long process. Second, the findings on gender roles also illustrate the value of approaching unequal gender norms by highlighting the benefits that can accrue from less rigid gender roles rather than challenging the gendered division of labour directly. Given the salience of gender norms around male provision, this research also suggests a role for novel programming that explicitly addresses the negative behaviours that arise from men's insecurity and frustration from their inability to provide. This would be particularly important in contexts where income generation activities are informal or unreliable, especially in highly commoditised urban settings where standards of living are closely related to access to resources. Third, the findings illustrate the importance of fostering an environment of non-tolerance of violence both to shift individual behaviour and to motivate sustained activism amongst community members. In the absence of sustained activism, social norms that define violence as a private issue, or normalise men's use of power over women, will likely be maintained. Community members will also not realise their potential role to challenge these norms. Fourth, the findings confirm the importance of including different levels of the Ecological Model in community-based IPV primary prevention programmes. Final, this research highlights the value of ensuring that programme implementers are able to provide good quality, transformative support to those seeking to reduce IPV and gender inequities, to ensure that those seeking help are effectively supported.
